# First-in-human study of WT1 recombinant protein vaccination in elderly patients with AML in remission: a single-center experience

**DOI:** 10.1007/s00262-022-03202-8

**Published:** 2022-04-27

**Authors:** Stefanie Kreutmair, Dietmar Pfeifer, Miguel Waterhouse, Ferenc Takács, Linda Graessel, Konstanze Döhner, Justus Duyster, Anna Lena Illert, Anna-Verena Frey, Michael Schmitt, Michael Lübbert

**Affiliations:** 1 Department of Internal Medicine I, Faculty of Medicine, Medical Center, University of Freiburg, 79106 Freiburg, Germany; 2 German Cancer Consortium (DKTK) and German Cancer Research Center (DKFZ), Partner site Freiburg, 69120 Heidelberg, Germany; 3 Center for Pathology, University Medical Center, University of Freiburg, 79106 Freiburg, Germany; 4 1st Department of Pathology and Experimental Cancer Research, Semmelweis University, 1085 Budapest, Hungary; 5 Department of Internal Medicine III, University Hospital, 89081 Ulm, Germany; 6 Department of Internal Medicine V, Hematology, Oncology, Rheumatology, University Hospital Heidelberg, 69120 Heidelberg, Germany; 7 German Cancer Consortium (DKTK) and German Cancer Research Center (DKFZ), 69120 Heidelberg, Germany

**Keywords:** WT1, Acute myeloid leukemia, AML, Protein vaccination, WT1 recombinant protein, Immune response

## Abstract

Wilms’ tumor 1 (WT1) protein is highly immunogenic and overexpressed in acute myeloid leukemia (AML), consequently ranked as a promising target for novel immunotherapeutic strategies. Here we report our experience of a phase I/II clinical trial (NCT01051063) of a vaccination strategy based on WT1 recombinant protein (WT1-A10) together with vaccine adjuvant AS01_B_ in five elderly AML patients (median age 69 years, range 63–75) receiving a total of 62 vaccinations (median 18, range 3–20) after standard chemotherapy. Clinical benefit was observed in three patients: one patient achieved measurable residual disease clearance during WT1 vaccination therapy, another patient maintained long-term molecular remission over 59 months after the first vaccination cycle. Interestingly, in one case, we observed a complete clonal switch at AML relapse with loss of WT1 expression, proposing suppression of the original AML clone by WT1-based vaccination therapy. Detected humoral and cellular CD4^+^ T cell immune responses point to efficient immune stimulation post-vaccination, complementing hints for induced conventional T cell infiltration into the bone marrow and a shift from senescent/exhausted to a more activated T cell profile. Overall, the vaccinations with WT1 recombinant protein had an acceptable safety profile and were thus well tolerated.

To conclude, our data provide evidence of potential clinical efficacy of WT1 protein-based vaccination therapy in AML patients, warranting further investigations.

**Supplementary Information:**

The online version contains supplementary material available at 10.1007/s00262-022-03202-8.

## Introduction

Acute myeloid leukemia (AML) is the most common hematologic malignancy in adults, characterized by increased proliferation of immature myeloid cells. An active involvement of the immune system in the pathogenesis of AML is observable, as the curative potential of allogeneic hematopoietic stem cell transplantation (allo-HSCT) is dependent on immunotherapeutic effects (graft-versus-leukemia effect) and not only on the ability to administer otherwise lethal doses of cytoreductive therapies [1]. The outcome of elderly patients, not eligible for intensive therapy due to comorbidities or reduced performance status, is particularly poor with a median survival of only 5–10 months [2–4]. Hence, novel and effective treatment strategies are urgently needed to overcome immune escape of AML cells.

The *Wilms' tumor 1 (WT1)* gene encodes a zinc finger motif-containing transcription factor involved in regulation of cell growth and differentiation [5–7]. There is much debate about the function of WT1 in malignancies, as there is evidence for *WT1* operating as both a tumor suppressor gene as well as an oncogene [8]. While *WT1* mutations occur in a proportion (10%) of AML patients, the gene is highly expressed in the majority of AMLs [9–11]. In leukemic cells, WT1 acts as a transcriptional activator of several oncogenes (e.g. *MDR1, BCL2A1*) [12, 13] and suppressor for the transcription of many tumor suppressor genes (e.g., *DMTF1, IRF8*) [14–16]. These data point to *WT1* as an oncogene in AML, supported by a correlation of *WT1* overexpression as well as *WT1* mutations with worsened overall survival (OS) and resistance to chemotherapy [17–20]. As there is no or only very low expression of *WT1* in normal hematopoietic cells, the overexpression of *WT1* exclusively in the leukemic clone qualifies the transcript as a “leukemic marker” useful in targeting these malignant cells [21]. Several therapeutic approaches describe WT1-based vaccination as a potential strategy to boost anti-cancer immunity by induction of leukemia-specific immune response [22–24]. There is increasing evidence for WT1-based vaccination strategies resulting in immunological and clinical benefit to AML patients [22, 25–29].

Different vaccination strategies use either target protein peptides (e.g., WT1_126-134_) or target protein-based recombinant proteins (e.g., first-ever reported WT1-A10 in this study) together with different adjuvants (e.g., AS01_B_) [22, 25–31]. The usage of recombinant proteins instead of peptides allows a potential immune response to multiple epitopes. Broadening the repertoire of the induced immune response, this vaccination strategy could be offered to all patients independent of HLA expression, since these recombinant proteins carry epitopes with binding motifs for several HLA molecules [32, 33].

Our results describe a single-center experience of a phase I/II study of first-in-human WT1 protein-based vaccination (WT1-A10 + AS01_B_) in patients with AML in complete remission with incomplete blood count recovery or partial remission post-induction therapy. We report here on clinical benefit, humoral and cellular immune response of treated AML patients as well as safety profile and tolerability of WT1 protein-based vaccination therapy.

## Material and methods

### Patients

Patients eligible for the study were required to have de novo or secondary AML as defined by World Health Organization (WHO) classification. Further eligibility criteria included expression of WT1 transcripts in AML blasts at initial diagnosis, detected by quantitative RT-PCR. Eligible patients received at least one (> 60 years old) or two (≤ 60 years old) induction chemotherapy treatments according to the institution’s standard of care. The AML had to have responded with partial remission (PR) or morphologic complete remission with incomplete blood count recovery (CRi). At time of enrollment, Eastern Cooperative Oncology Group (ECOG) performance status had to be 0, 1 or 2 and there had to be an adequate hepatic and renal function defined as serum bilirubin < 1.5 times the Upper Limit of Normal (ULN), serum ALT < 2.5 times the ULN as well as calculated creatinine clearance > 50 ml/min.

Exclusion criteria were acute promyelocytic leukemia and patients who had received or were to receive allogeneic HSCT. Among other exclusion criteria, patients who had received fludarabine, clofarabine or cloretazine within 12 months, or showed hypercalcemia, symptomatic autoimmune disease (except vitiligo) or were known to be HIV-positive were excluded from the study.

The study was initiated and sponsored by GlaxoSmithKline Biologicals SA (NCT01051063). The study was approved by the Local Ethics Committee of the University Hospital Freiburg, and written informed consent was obtained from each patient prior to study entry. Patients were treated between June 2011 and September 2015. Due to a change in corporate alignment, the study was prematurely terminated by the sponsor.

### Vaccination protocol

The vaccine consisted of WT1-A10, a truncated WT-1 protein retaining the N-terminus (amino acids 2–281) of full length WT1 protein (429 aa) linked to the first 11 amino acids of trimethylamine N-oxide reductase signal peptide via one histidine residue combined with the liquid AS01_B_ adjuvant. AS01_B_ is an Adjuvant System containing MPL (3-*O*-desacyl-4´-monophosphoryl lipid A [produced by GSK]), QS-21 (*Quillaja saponaria* Molina, fraction 21 [licensed by GSK from Antigenics LLC, a wholly owned subsidiary of Agenus Inc., a Delaware, USA corporation]) and liposome (50 µg MPL and 50 µg QS-21). One human dose of WT1-A10 + AS01_B_ contained 200 μg of WT1-A10 antigen. Patients received the vaccine by intramuscular injection.

The first vaccine administration had to be given within 70 days (ten weeks) after the last chemotherapy administration. Vaccination schedule included cycle 1 (6 doses, each given at 2-week intervals), cycle 2 (6 doses, each given at 3-week intervals), cycle 3 (4 doses, each given at 6-week intervals) and cycle 4 (4 doses, each given at 3-month intervals) followed by 4 doses each given at 6-month intervals.

Routine toxicity assessments were conducted throughout the trial and were graded in accordance with the National Cancer Institute Common Toxicity Criteria for Adverse Events (CTCAE) version 3.0. Evidence of disease progression was evaluated regularly using peripheral blood measurements and bone marrow aspirates/ biopsies including measurable residual disease (MRD) assessments.

### Measurement of MRD and WT1 expression

DNA and total RNA were extracted from peripheral blood or bone marrow samples using the Qiasymphony miniDNA kit and the Qiasymphony RNA kit, respectively, according to the manufacturer’s instructions (Qiagen, Hilden, Germany). DNA isolation protocol included RNase I for removing RNA from DNA preparations. RNA and DNA were quantified with the NanoDrop spectrophotometer (Thermo Scientific, Dreieich, Germany). Random hexamer oligonucleotides were used to prime cDNA synthesis by RT from a minimum of 1 μg RNA template using the Maxima cDNA first strand kit (Thermo Scientific, Dreieich, Germany). Molecular targets for MRD assessment were analyzed by digital PCR using the QX200 Droplet Digital PCR system according to the manufacturer’s protocol (Bio-Rad Laboratories, Munich, Germany). All samples were analyzed at least in duplicates. Each reaction mixture was partitioned into approximately 20,000 droplets using a droplet generator (Bio-Rad Laboratories, Munich, Germany). Cycled droplets were read in a QX200 droplet-reader and the analysis of the dPCR data was performed using QuantaSoft analysis software (Bio-Rad Laboratories, Munich, Germany). The threshold for each molecular marker used for MRD detection was established as suggested by the Clinical and Laboratory Standards Institute (guideline EP 17 A2). Appropriate positive and negative controls were used for each molecular aberration. WT1 quantitative assessment was used as an additional MRD marker according to the assay developed by Cilloni et al. and adapted for dPCR [34].

### Humoral immune response

Specific anti-WT1 antibodies induced by the WT1-A10 + AS01_B_ vaccination were measured by ELISA using either the WT1-A10 recombinant protein or WT1 derived peptides spanning the entire WT1 sequence as coating antigen. The results were provided in EU/ml (Suppl. Table 1). A patient was considered as seropositive if the antibody titer was superior or equal to the assay cut-off.

### T cell immune response

Peripheral blood mononuclear cells (PBMCs) were cultured for 14 days in 24 independent micro-cultures in limiting dilution conditions (2 × 10^5^ cells/well), with antigen-specific stimulation (protein immunocomplexed with pool of plasma containing anti-WT1 antibodies) and with IL-2 and IL-7. On day 14, PBMC micro-cultures were divided in two to allow antigen-specific and antigen non-specific (irrelevant) re-stimulation. Antigen-specific stimulation was performed with a pool of 123 15mer peptides (with an overlap of 10 amino acids [aa]) covering the entire WT1 (1 μg/ml/peptide). Irrelevant re-stimulation was performed with a pool of 43 15mer peptides derived from the NY-ESO-1 protein, plus negative control peptide (aa sequence: NEGATIVESQNTRQL) to ensure equivalent total peptide mass in both specific and irrelevant stimulation conditions. CD4^+^ and CD8^+^ T cells in each well were assessed by intracellular flow cytometry for their ability to produce both IFN-γ and TNF upon antigen stimulation. Intracellular cytokine staining was performed with the following antibodies: IFN-γ-FITC, CD3-PercP, TNF-PE-Cy7, CD4-APC-H7 and CD8-V450. Results were analyzed using FlowJo software (Becton Dickinson). Frequencies of antigen specific T-cell precursors (assuming a clonal response) were computed as follows: for each pair of wells the ratio between the percentage of positive cells in the specific and irrelevant stimulation was calculated. The geometric mean of the 24 ratios (GMR, considered as an immunogenicity score) was calculated to integrate the average responses observed in the 24 independent wells. For wells with fewer than 50 positive antigen-specific events the well ratio was set at 1. GMR cut-offs were calculated for both CD4^+^ and CD8^+^ T cells from 23 healthy donors using the same analysis templates, and values were determined as 2.68 (for CD4^+^ T cell analysis) and 1.15 (for CD8^+^ T cell analysis). A patient was considered as an immune responder if the GMR after vaccination was both above the cut-off and at least four times higher than the GMR at baseline.

### Clonal heterogeneity assessment

The Illumina TruSight Myeloid Sequencing Panel was used for targeted resequencing and processed as described by the manufacturer (Illumina Inc., San Diego, CA). FASTQ-files were further analyzed with the SeqNext software (JSI Medical Systems, Ettenheim, Germany). We used a significance threshold of 1–5% for the detection of missense mutations, with a minimum coverage of 200 reads and 20 reads per variant.

### High-dimensional single-cell cytometry

PBMCs were thawed, resuspended in cell culture medium supplemented with 2U ml − 1 benzonase. Cell count was calculated using an automated cell counter (Bio-Rad). Subsequent procedure was performed as described previously [35]. 1 million (mio) cells per sample were directly stained for cytometry analysis (surface panel), while 1 mio cells were restimulated with 50 ng ml − 1 phorbol 12-myristate 13-acetate (Sigma–Aldrich) and 500 ng ml − 1 ionomycin (Sigma–Aldrich) in the presence of 1 × Brefeldin A and 1 × Monensin (both BD Biosciences) for 5 h at 37 °C. For surface staining, cells were incubated in Live/Dead Fixable Blue mixture (Thermo Scientific, 1:500), followed by a blocking step to avoid nonspecific binding (True Stain FcX (BioLegend)). Anti-human flow cytometric antibodies were purchased pre-conjugated (Suppl. Table 2) and used for surface staining step (15 min at 37 °C). For intracellular cytometry, after surface-antibody labeling, cells were fixed and permeabilized using Cytofix/Cytoperm reagent (BD Biosciences). Intracellular labeling was then performed in 1 × permeabilization buffer (Thermo Scientific) for 12 h at 4 °C using the antibodies described in Suppl. Table 3. Flow cytometry samples were acquired on a Cytek Aurora (Cytek Biosciences). Quality control of the Cytek Aurora was performed daily as instructed by the manufacturer. For downstream analysis, dead cells and doublets were excluded using FlowJo (TreeStar). Samples with viability lower than 20% and fewer than 500 live, CD45 positive cells were excluded. Cytometry data were transformed with an inverse hyperbolic sine (arcsinh) function using the R environment (range 150–20,000). To balance the influence of markers with different dynamic ranges, we performed channel-based percentile normalization using the 99.9th percentile of each marker across the whole dataset [36]. Two-dimensional UMAP (Uniform Manifold Approximation and Projection) projections were calculated using the umap package [37]. All FlowSOM-based clustering was performed on the whole dataset, and the results were overlaid on the dimensionality reduction maps [38]. All plots were drawn using ggplot2.

### Immunohistochemistry of bone marrow biopsies

Bone marrow biopsies were fixed in 4% buffered formalin (FA). After fixation, all biopsies were subjected to decalcification in a mixture of 10% ethylenediaminetetraacetic acid disodium salt (Serva) and 3.3% tris-(hydroxymethyl) aminomethane (AppliChem) in dd H2O at a pH of 7.0 to 7.2 overnight and then subsequently embedded in paraffin. Serial 2-µm sections were deparaffinized in xylene and graded alcohols, followed by specific antigen retrieval in target retrieval solution (pH 9) in a steamer (Dako, Glostrup, Denmark; 4–6 min for FA-fixed biopsies, depending on the antibody lot). After incubation with 1 of 2 primary antibodies for 1 h at room temperature (CD4 and CD8 [DAKO], RTU.) staining was detected with the Dako EnVision FLEX Visualization System (Dako EnVision FLEX, High ph(Link) CODE K8000). The reactivity of each antibody was tested with appropriate control tissues (e.g., tonsil, appendix, bone marrow, tumors) as used routinely by the Laboratory of Immunohistochemistry of the Department for Pathology, University Medical Center Freiburg. The sections were counterstained with hematoxylin (Waldeck) and mounted. The percentage (mean ± SD) of CD4 or CD8 positive T lymphocytes of all nucleated precursor cells was determined by counting a maximum of 500 cells / biopsy using random high-power fields (600 × original magnification). Cells that were both cytoplasmic- and membrane-positive were regarded as positive. Every biopsy was evaluated independently by two persons, both blinded to the diagnosis and time of treatment.

### Statistical analysis

A Wilcoxon signed rank test was used for statistical analysis, unless otherwise stated. GraphPad Prism version 6.07 was used to perform and visualize statistical analysis (La Jolla, CA). A *p* value < 0.05 was determined to be statistically significant.

## Results

### Clinical baseline characteristics of the patients

A total of five patients (4 males, 1 female) were enrolled on the WT1 protein-based vaccination study in our institution (Table 1). Three patients were diagnosed with de novo AML (patients #1,2,3), one patient with secondary AML from CMMoL (patient #4) and one patient with therapy-related AML after treatment for B cell chronic lymphocytic leukemia (B-CLL) (patient #5). The median age was 69 years (range, 63–75 years) and ECOG performance status ranged from 0 to 2. Four out of five patients had a normal karyotype (#1,3,4,5) while patient #2 was diagnosed with 46,XY,del(7)(q22q36). Further molecular testing demonstrated three patients with *NPM1* mutation (#1,3,5) and one patient with *FLT3* internal tandem duplication (*FLT3-ITD*) mutation (#4). The clonal heterogeneity was assessed by next generation sequencing of 54 genes frequently mutated in myeloid malignancies (Table 1). Thus, enrolled patients showed different AML clones and harbored at least three different genetic aberrations (patients #1,3,5) and up to seven aberrations (patient #4). Especially patient #4 seemed to have different AML subclones as the variant allele frequency (VAF) of measured aberrations ranged from 9.3 to 49%. Using cytogenetic and molecular characteristics, risk assessment was stratified according to the European LeukemiaNet (ELN) 2010 prognostic scoring system [39]: three patients (#1,3,5) showed favorable, one (#4) intermediate I and one (#2) intermediate II risk AML. All five patients received at least one anthracycline-cytarabine-based induction regimen and three patients received post-remission treatment with two (patient #2,3) or three consolidation cycles (patient #1), respectively [40]. Thus, all patients completed planned upfront antileukemic therapy prior to study enrollment and achieved CRi (patient #1,3,4,5) or cytogenetic relapse with del(7q) positive clone (#2) according to standard criteria (Tables 1 and 2).

**Table 1:** Baseline characteristics of patients

Pat. No	Age, y/ gender	ECOG	Diagnosis	Genetics	Karyotype	ELN 2010 risk	AML therapy	Disease status baseline
1	63/ m	0	de novo AML	*IDH2* (c.419G > A; VAF 48%) *NPM1* (c.860_863dupTCTG; VAF 35%) *SRSF2* (c.284C > T; VAF 42%)	46,XY	Favorable	A-ICE Induction × 2, Consolidation × 3 (AMLSG 09–09)	CRi
2	75/ m	1	de novo AML	*ASXL1* (c.1934dupG; VAF 36%) *EZH2* (c.2044G > A; VAF 76%) *IDH1* (c.394C > T; VAF 35) *IKZF1* (c.556G > T; VAF 41%) *TET2* (c.4537 + 1G > A; VAF 44%) *U2AF1* (c.470A > G; VAF 46%)	46,XY del(7) (q22q36)	Intermediate II	MiCE Induction, Mini-ICE Consolidation × 2	Hematol. CR, cytogenetic relapse
3	72/ f	1	de novo AML	*FLT3#* (c.2516A > G; VAF 39%) *NPM1* (c.860_863dupTCTG; VAF 29%) *SRSF2* (c.284C > G; VAF 41%)	46,XX	Favorable	MiCE Induction, Mini-ICE Consolidation × 2	CRi
4	69/ m	2	sAML (from CMMoL)	*ASXL1* (c.1934dupG; VAF 37%) *DNMT3A* (c.2645G > A; VAF 46%) *FLT3-ITD* (allelic ratio 0.573) *IDH2* (c.419G > A; VAF 49%) *NRAS* (c.35G > A; VAF 9.3%) *RUNX1* (c.160G > T; VAF 11%; c.611G > A; VAF 50%) *SF3B1* (c.2098A > G; VAF 48%)	46,XY	Intermediate I	MiCE Induction (single course)	CRi
5	65/ m	2	t-AML (from B-CLL)	*DNMT3A* (c.2311C > T; VAF 32%) *KRAS* (c.35G > C; VAF 27%) *NPM1* (c.860_863dupTCTG; VAF 20%)	46,XY	Favorable	A-ICE Induction (single course) (AMLSG 09–09)	CRi

Clinical baseline characteristics of patients treated with WT1 protein vaccination. *Pat*.*No*. Patient number. ^#^Variant of uncertain significance, likely pathogenic

**Table 2:** Clinical course

Pat. No	No. of Vacc.	Duration of Vacc. (mon)	PFS from 1st Vacc. (mon)	OS from 1st Vacc. (mon)	Blasts in PB prior to Vacc. (%)	Blasts in BM prior Vacc. (%)	Blasts in PB after Vacc. (%)	Blasts in BM after Vacc. (%)	NPM1 MRD NCN in BM prior to Vacc	NPM1 MRD NCN in BM at best response	NPM1 MRD NCN in BM at relapse	WT1 NCN in BM at diagnosis	WT1 NCN in BM prior to vacc	WT1 NCN in BM at best response	WT1 NCN in BM at relapse
1	20	24	59	> 75	0	0	0	0	0.00	0.00	29.60	1049.0	n.a	11.7	173.6
2	3	1	1	10	0	4	9	8				293.0	704.0		
3	18	18	18	24	0	2	0	18	0.00	0.00	0.45	5023.0	n.a		2.6
4	3	1	1	3	3	4	18	n.a				1163.0	1479.0		
5	18	20	65	65	0	0	0	0	4.43	0.00	no relapse	308.0	n.a	20.4	no relapse

Table demonstrating number of vaccination cycles, blast percentages, MRD and *WT1* values during the clinical course of therapy. *NCN* Normalized copy numbers. *Vacc.* Vaccination. *mon*. Month. *PFS* Progression-free survival. *OS* Overall survival. *n.a* Not applicable

### Good tolerability of WT1 vaccination therapy

Routine toxicity assessments were conducted throughout the vaccination therapy on all five patients enrolled in the trial. WT1-based vaccination proved safe and well-tolerated. There were no serious adverse events in relationship to vaccination therapy. Overall, only two out of the five patients experienced therapy-related toxicity without restrictions in their quality of life (Table 3). Specifically, patient #3 once showed injection site pain (CTCAE grade 2) and patient #5 once demonstrated injection site inflammation (CTCAE grade 1). Symptoms were of mild/ moderate severity and resolved completely. No hematologic toxicity was noted.

**Table 3:** (Serious) adverse events

Pat. No	AE/ SAE	Event	Max intensity (CTCAE grade)	Outcome	Relationship to investigational product
1	AE	iron overload	1	not recovered/ resolved	No
1	AE	headache	1	recovered/ resolved	No
1	AE	dizziness	1	recovered/ resolved	No
1	AE	infection of the upper respiratory tract	1	recovered/ resolved	No
1	AE	joint pain	1	recovered/ resolved	No
1	AE	heart burn	1	recovered/ resolved	No
1	AE	bronchial infection	2	recovered/ resolved	No
1	SAE	infection with fever	3	not recovered/ resolved	No
2	SAE	diverticulitis	3	recovered/ resolved	No
2	AE	anemia	3	recovered/ resolved	No
3	AE	headache	2	recovered/ resolved	No
3	AE	nausea	1	recovered/ resolved	No
3	AE	injection site pain	2	recovered/ resolved	Yes
3	AE	diarrhea intermittent	1	recovered/ resolved	No
3	AE	fever	1	recovered/ resolved	No
4	SAE	dyspnea	3	recovered/ resolved	No
4	SAE	leukocytosis	4	recovered/ resolved	No
4	SAE	pneumonia	3	recovered/ resolved	No
5	AE	verrucosis	1	not recovered/ resolved	No
5	AE	pruritus both legs	1	recovered/ resolved	No
5	AE	arrhythmia	1	recovered/ resolved	No
5	AE	depression mental	1	not recovered/ resolved	No
5	AE	injection site inflammation	1	recovered/ resolved	Yes
5	AE	syncope	3	recovered/ resolved	No
5	SAE	bronchitis	3	recovered/ resolved	No
5	SAE	bronchitis	3	recovered/ resolved	No
5	SAE	pneumonia	2	recovered/ resolved	No
5	SAE	fever	2	recovered/ resolved	No
5	AE	recurrence CLL	1	not recovered/ resolved	No
5	SAE	hypercalcemia	4	recovered/ resolved	No
5	AE	syncope	3	recovered/ resolved	No
5	AE	chill	1	recovered/ resolved	No

Table demonstrating (serious) adverse event during the clinical course of therapy. *AE* Adverse event. *SAE* Serious adverse event

### Clinical efficacy

#### Patients 2, 4: Early AML relapse after 3 vaccinations

Patient #2 started WT1-based vaccination treatment with a cytogenetic relapse after three courses of chemotherapy, while patient #4 received sole induction therapy, thereby achieving CRi prior to vaccination. Both had to stop vaccination therapy after three doses due to overt AML relapse. Patient #2 showed rising numbers of blasts in bone marrow (8%) and peripheral blood (9%) as did patient #4 with 18% of blasts in differential blood count (Table 2).

After receiving hypomethylating agents, patient #2 showed a stable course for another 8 months before he died due to AML progression 10 months after the first vaccination cycle. Of note, the deletion (7)(q22q36) was present with 80% frequency prior to vaccination and showed a frequency of 7.5% after vaccination and HMA therapy. These results may indicate a clonal suppression due to WT1-based vaccination or HMA therapy although the patient did not develop a humoral immune response: anti-WT1 IgG was below the cut-off value at day 35 and four months later (Suppl. Table 1).

The AML of patient #4 seemed to be even more aggressive. Due to AML relapse, the patient had to stop vaccination therapy and died due to infection three months after the first WT1-based treatment injection. Of note, also patient #4 failed to develop a humoral immune response (measured at day 35; Suppl. Table 1).

#### Patient 1: 59-months remission maintenance with 20 vaccinations (following 5 chemotherapy courses)

**Fig. 1:**
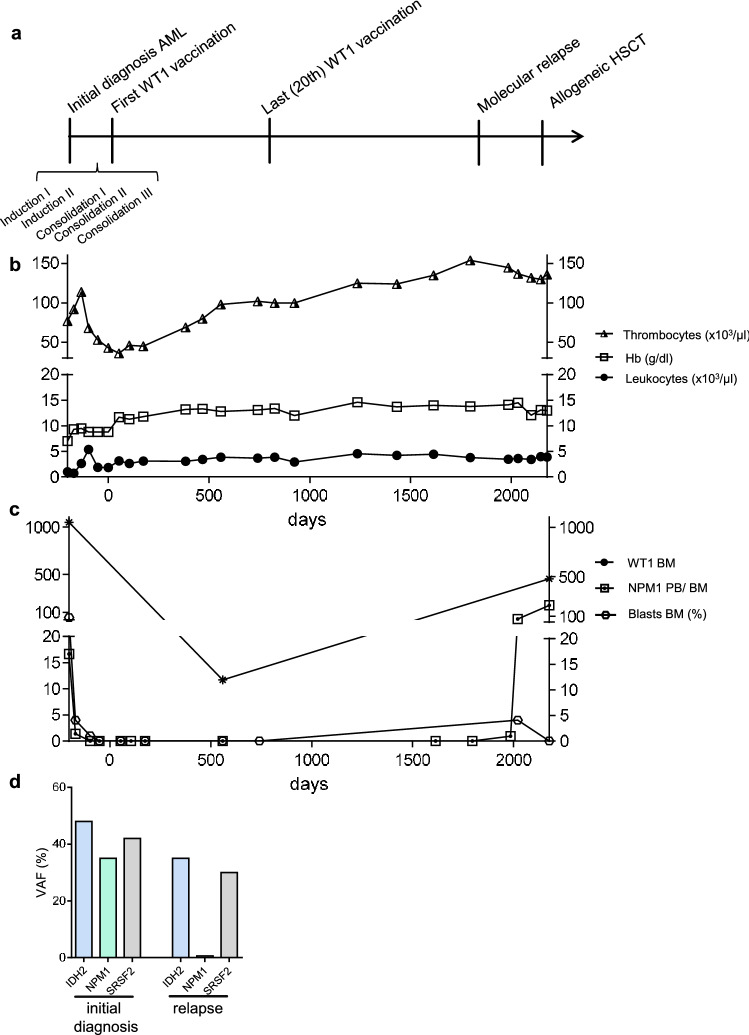
Patient #1 **a** Timeline demonstrating initial diagnosis, status of remission and therapeutic interventions. **b** Leukocytes, thrombocytes and hemoglobin values during clinical course. **c** Frequencies of blasts, *NPM1* and *WT1* normalized copy numbers (NCN) in bone marrow during clinical course. Peripheral blood was used for the measurement of *NPM1* NCN at day 384, 468, 825, 923, 1236, 1432, 1614, 1796 and 1985. **d** Gene mutations at initial diagnosis and relapse of AML measured by next-generation-sequencing (54-myeloid-gene panel)

After five courses of chemotherapy, patient #1 underwent 20 WT1-based vaccine injections over a period of 24 months and showed disease control of 59 months duration (Table 2, Fig. 1a–d). The blood counts were stabilizing during the time of vaccination, especially the thrombocytes were rising to low normal values (Fig. 1b). At the beginning of vaccination therapy, the patient started with AML being in molecular remission. While bone marrow did not show increased blast counts (Fig. 1c) over the time of vaccination, he represented a smoldering molecular relapse of AML measured in the peripheral blood 11 months after the last WT1 vaccination (Fig. 1c). Bone marrow aspirate showed the AML clone harboring *NPM1*, *IDH2* and *SRSF2* mutations, which had already been determined at initial diagnosis (Fig. 1d). With a variant allele frequency of 0.56%, the clone harboring *NPM1* seemed to be minor at the time of relapse. Thereupon, *NPM1* normalized copy numbers (NCN) were slowly rising over 12 months, when the patient underwent allogeneic stem cell transplantation and is now in CR.

#### Patient 3: 18-months remission maintenance with 18 vaccinations (following 3 chemotherapy courses)

**Fig. 2:**
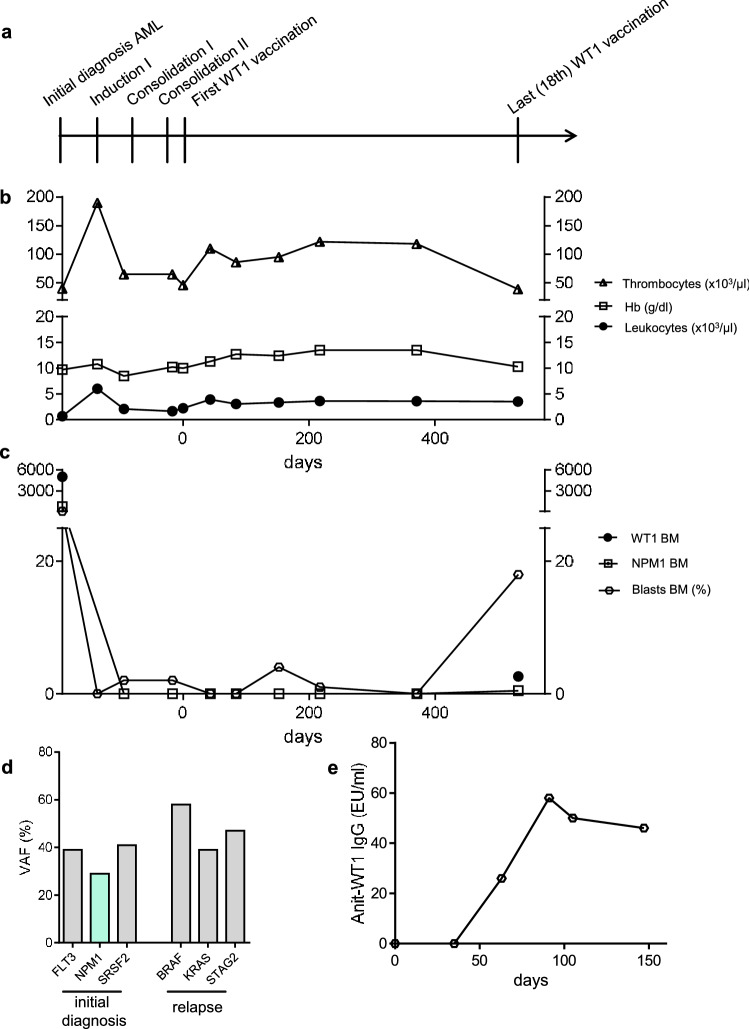
Patient #3 **a** Timeline demonstrating initial diagnosis, status of remission and therapeutic interventions. **b** Leukocytes, thrombocytes and hemoglobin values during clinical course. **c** Frequencies of blasts, *NPM1* and *WT1* NCN in bone marrow during clinical course. **d** Gene mutations at initial diagnosis and relapse of AML measured by next-generation-sequencing (54-myeloid-gene panel). **e** Anti-WT1 IgG antibody response measured by ELISA

Patient #3 received three courses of chemotherapy prior to WT-based vaccination and showed above-average CR and survival duration (Table 2). Nevertheless, after 18 injections of WT1-based vaccination therapy and after more than one year of molecular remission, the patient showed slightly dropping thrombocyte counts (Fig. 2a, b). AML relapse (18% blasts in bone marrow; Fig. 2c) was diagnosed 18 months after the first vaccination cycle. The patient underwent hypomethylating therapy, but died five months later due to infection.

#### Patient 5: Complete and sustained MRD clearance with 18 vaccinations (following a single chemotherapy course)

**Fig. 3:**
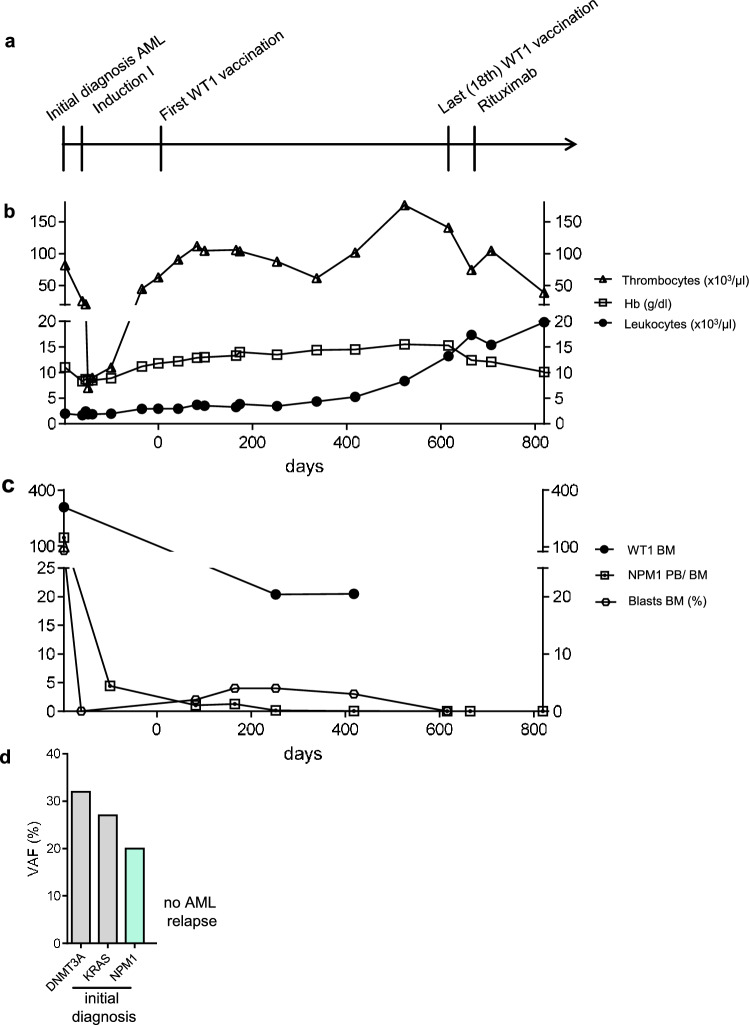
Patient #5, clinical course **a** Timeline demonstrating initial diagnosis, status of remission and therapeutic interventions. **b** Leukocytes, thrombocytes and hemoglobin values during clinical course. **c** Frequencies of blasts, *NPM1* and *WT1* NCN in bone marrow during clinical course. *NPM1* NCN at day + 665 and + 819 were measured from peripheral blood. **d** Gene mutations at initial diagnosis of AML measured by next-generation-sequencing (54-myeloid-gene panel)

**Fig. 4:**
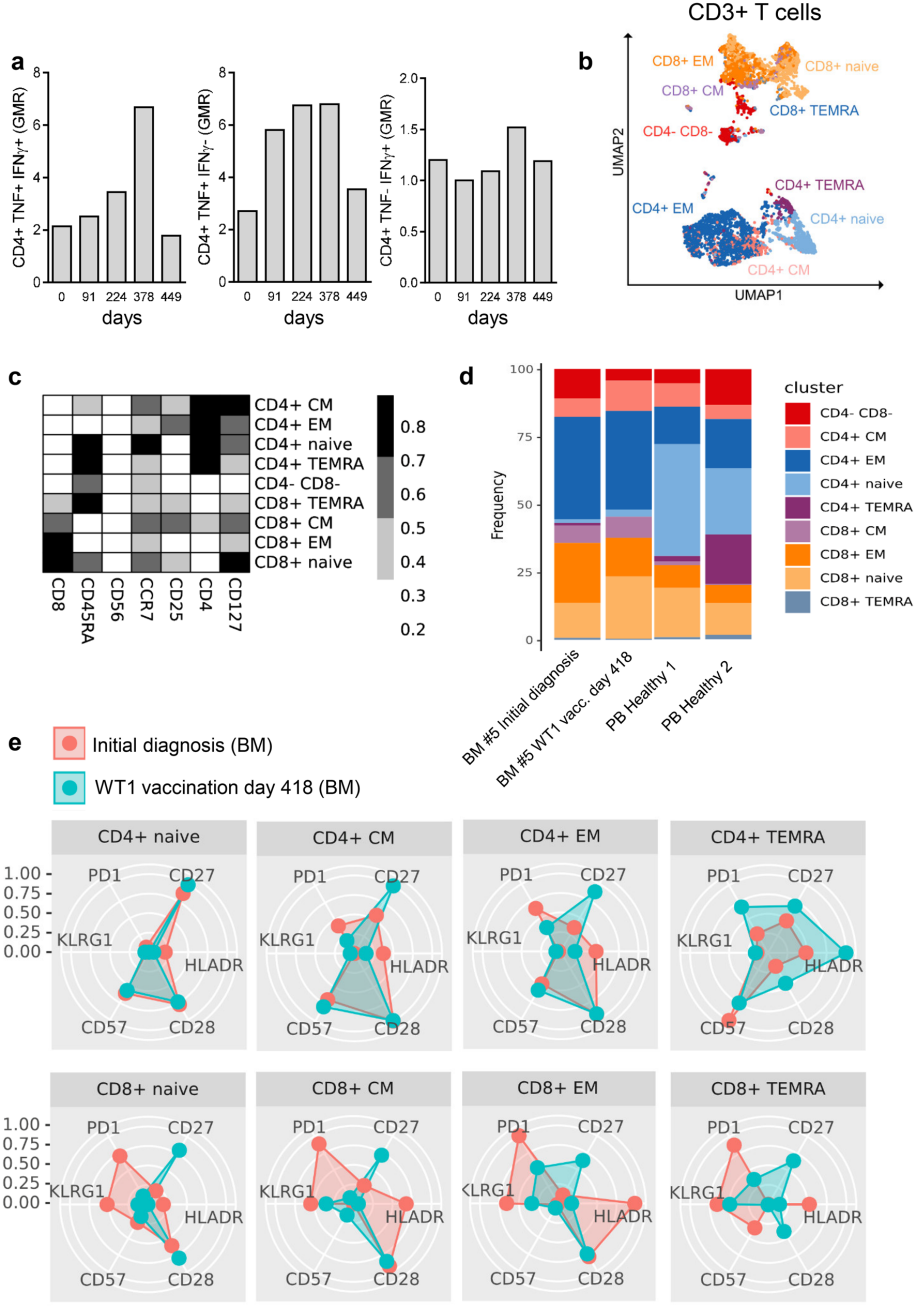
Patient #5, T cell response **a** Specific CD4 + T cell response measured by flow cytometry. **b** UMAP with FlowSOM overlay showing the total CD3^+^ T cell compartment of combined samples. 1000 cells were subsetted from every sample. **c** Heatmap depicting median expression of various markers in FlowSOM-derived clusters shown in (**b**). **d** Barplot depicting the frequency of FlowSOM-derived T cell subsets of all CD3^+^ T cells in indicated samples. **e** Radar chart depicting median expression of indicated markers in FlowSOM-generated T cell subsets. BM samples from patient #5 at initial diagnosis and during WT1 vaccination (day 418) are shown

**Fig. 5:**
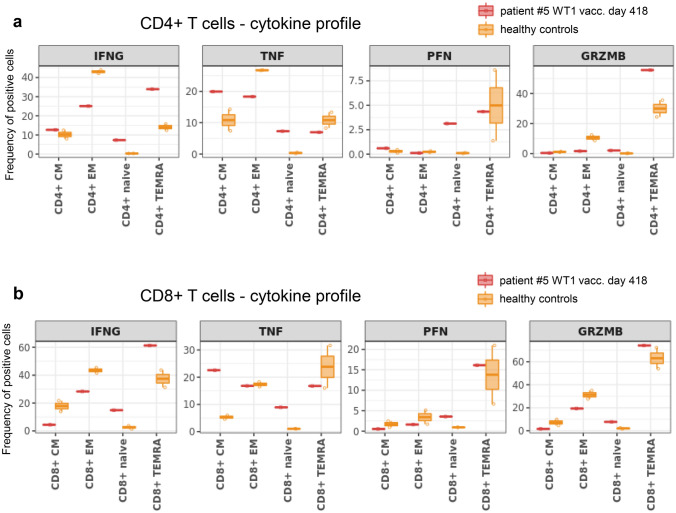
Patient #5, T cell cytokine profile **a** and **b** Box plots depicting median frequency and 25th and 75th percentile of cytokine positive cells in indicated FlowSOM-generated T cell subsets of patient #5 and healthy controls **a** for CD4^+^ T cells and **b** for CD8^+^ T cells

After a single course of chemotherapy, patient #5 underwent 18 vaccinations over a period of 20 months and showed no signs of AML relapse during this time period (Table 2, Fig. 3a–d). The patient demonstrated stable blood values (Fig. 3b) and normal blast counts (Fig. 3c). Interestingly, he showed decreasing measurable residual disease (MRD) (*NPM1* NCN) during vaccination therapy, resulting in molecular CR at visit for the 18th vaccination (Fig. 3c and d). Although the patient demonstrated no specific anti-WT1 humoral response (anti-WT1 IgG below cut-off of 7 EU/ml day 0–448; Suppl. Table 1) or CD8^+^ T cell response (Suppl. Figure 1a), a WT1-specific CD4^+^ T cell response was detected using flow cytometry (Fig. 4a), with a cytokine polarization profile focused on TNF production rather than IFN-γ. Using high-dimensional single-cell cytometry (Fig. 4b–e, Suppl. Figure 1b, Suppl. Table 2 and 3), patient #5 showed high frequencies of CD8^+^ central and effector memory cells, as well as CD4^+^ effector memory cells, both at initial diagnosis and at day 418 of WT1 vaccination (Fig. 4d). Interestingly, the T cell subpopulations changed their profile when comparing initial diagnosis vs. day 418 of WT1 vaccination (Fig. 4e). WT1 vaccination seemed to induce or at least support a shift from exhausted (less PD-1) and senescent (less KLRG1, less CD57 (T effector memory CD45RA^+^ cells (TEMRA)), higher CD28 (TEMRA)) cells to more (early) activated T cells (higher levels of co-stimulatory molecule CD27 and CD28). Regarding the overall cytokine polarization profile (not only specific to WT1; Fig. 5a,b, Suppl. Figure 2, Suppl. Table 2 and 3), the T cells of patient #5 during WT1 vaccination showed no difference in perforin levels, but demonstrated higher frequencies of granzyme B positive TEMRA cells – both in the CD4 and CD8 fraction – as a sign of cytotoxicity.

20 months after the first WT1-based vaccination, his B-CLL progressed. The patient died from B-CLL acceleration after several therapy lines for CLL 5.5 years after initial diagnosis of AML and 3.5 years after the last WT1 vaccination with persistent CR of AML on a cytological and molecular level.

### Overall outcome and T cell response

Summarizing the overall outcome, with a median progression-free survival (PFS) of 28.8 months (range 1–59 months) and median OS of 35.4 months (range 3–75 months) from beginning of vaccination therapy, this older patient cohort (median age 68.8 years) showed above-average clinical outcome (Table 2). A median OS of 42.2 months (range 8–82 months) from initial diagnosis of AML points to a potential clinical efficacy of WT1-based vaccination therapy.

**Fig. 6:**
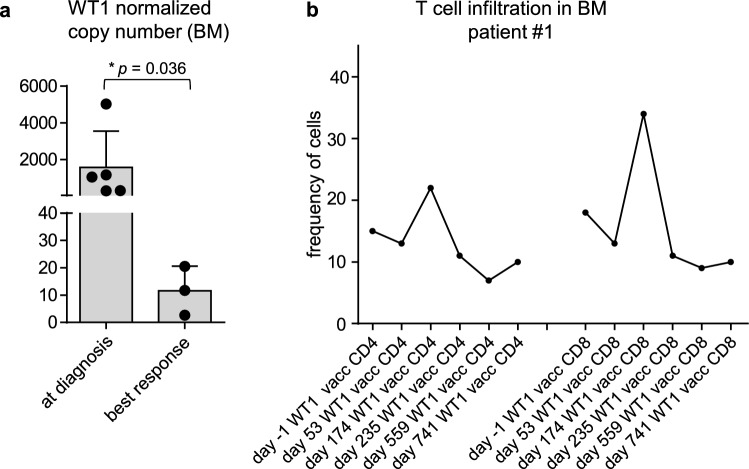
*WT1* expression and T cell infiltration **a** Diagram demonstrating *WT1* NCN in BM at initial AML diagnosis and best response during WT1 vaccination **b** Diagrams demonstrating the frequency of indicated T cell populations of all nucleated precursor cells before and after WT1-based vaccination in the BM measured by IHC, shown for patient #1

While all vaccinated patients showed high *WT1* NCN in the bone marrow before WT1-based vaccination therapy, the mean *WT1* NCN were lower after vaccination (Table 2, Fig. 6a). Furthermore, in all analyzed patients (#1,2,3,5), we detected an increase in frequency of CD4^+^ T cells in the bone marrow after WT1-based vaccination (Fig. 6b, Suppl. Figure 3). Same applied to CD8^+^ T cell frequency – except for patient #2 who showed early relapse. These results are pointing to the efficacy of WT1-based vaccination in those patients by T cell infiltration into the bone marrow and shaping their immune profile.

### Complete switch in clonal architecture and loss of *WT1* overexpression at relapse (patient 3)

Although patient #3 represented above-average CR duration while receiving WT1 protein-based vaccinations, relapse of AML was detected after more than one year of molecular response, with a complete clonal switch: the clone at initial diagnosis harbored *FLT3*, *NPM1* and *SRSF2* mutations, while at the time of relapse, *BRAF*, *KRAS* and *STAG2* mutations were detected (Fig. 2d). These results are pointing to an ongoing suppression of the *WT1* expressing AML clone. In line with this, an anti-WT1 antibody response was documented (Fig. 2e, Suppl. Table 1) and *WT1* NCN were low at the time of leukemia relapse (Fig. 2c), whereas patient #1, who relapsed with the same AML clone, showed increased *WT1* NCN in bone marrow aspirates (Fig. 1c).

## Discussion

A phase I/II study has been initiated to analyze the treatment effect and toxicity of WT1 protein-based vaccination in AML patients. Here, we further explored and report on this analysis of the five elderly patients with AML from our institution receiving a total of 62 vaccinations, three of who achieved above-average outcome in the absence of significant toxicity.

Several therapeutic strategies based on WT1 vaccines have been reported. Potential therapeutic activity has been described by both specific immunological and clinical responses [26, 29, 41–43]. A wide range of outcomes stems from several different vaccination approaches with a variety of doses, adjuvants, injection sites and application intervals as well as disease status at baseline [22, 44]. However, the majority of studies demonstrated clinical outcome being superior to historical data. Keilholz et al. reported on 10 out of 17 enrolled AML patients demonstrating stable disease, with four patients showing a blast reduction of more than 50% and two hematologic improvements [26]. One patient achieved CR after initial progression [25]. These results are particularly intriguing as the treated cohort mainly (13/19) showed progressive disease at baseline, with a median blast count of 45% [26]. Maslak et al. reported a median disease-free survival of 16.9 months and median OS estimated to be ≥ 67.6 months in a cohort of 22 AML patients in CR after induction therapy receiving multivalent WT1 peptide vaccine [29]. The median age of these patients was 64 years, thus a somewhat younger cohort compared to the five patients treated in our institution (median age 69 years). This fact–among others–could explain the benefit being even better than seen in the five patients treated at our institution. Comparable to above-average AML control in patient #1, #3 and #5 with OS rates of > 82, 30 and 72 months, long-term remission of more than 8 years was described in one AML patient treated with WT1 peptide vaccine by Tsuboi et al. [27]. Unfortunately, there was no possibility to rechallenge patient #1 to WT1-based vaccination at time of AML relapse. As the progress occurred 11 months after the last vaccine injection, this approach would have been potentially helpful, especially as the rising AML clone was diagnosed with the same characteristics including high *WT1* expression levels as also detected at initial diagnosis.

Furthermore, it is remarkable that the effect of WT1-based vaccination resulted in MRD clearance (*NPM1* NCN) in patient #5. This clonal suppression lasted even after B cell depletion (Rituximab) due to treatment of B-CLL. While the vaccine seemed to target the AML clone, the B-CLL was not affected by this immunotherapy. Of note, there was no *WT1* expression detectable in the CLL clone (data not shown). Refractory B-CLL but not AML was the cause for the patient's death 3.5 years later. Emphasizing the potential treatment effect of WT1 protein-based vaccination, patient #3 showed not only above-average response duration but also a clonal evolution due to potential selection pressure during vaccination therapy. The rising clone at AML relapse showed a completely different genetic profile and low *WT1* expression levels. To our knowledge, this is the first report of a complete clonal switch under WT1-based vaccination, which further supports the specificity of this immunotherapeutic approach.

Both patient #2 and #4 had an AML relapse already after three vaccine injections. Patient #2 showed cytogenetic relapse at the beginning of vaccination and patient #4 was diagnosed with secondary AML arising from CMML, facts which could also contribute to inferior outcome compared to patients #1, 3 and 5. Repeated PR1 and WT1 peptide vaccination failed to induce sustained high-avidity, epitope-specific CD8^+^ T cells in myeloid malignancies [42]. In multivalent WT1 vaccinated patients, CD8^+^ T cell responses represented by IFN-γ secretion as well as specific CD4^+^ T cell proliferation were reported after 6 or 12 vaccinations [29]. In comparison, patient #5 demonstrated a CD4^+^ T cell response with a maximum after 16 vaccinations and high percentage of TNF and IFN-γ double positive lymphocytes, pointing to a high effector potential of these cells.

Our data points to the efficacy of WT1-based vaccination by conventional T cell infiltration into the bone marrow and shaping their immune profile–toward less exhaustion and more activated patterns. However, we were not able to verify a specific cellular immune response in the CD8^+^ T cell compartment – at least in the one patient measured, although the recombinant WT1 protein carried several potential CD4^+^ and CD8^+^ epitopes. Thus, the strong CD4^+^ T cell activation together with the lack of CD8^+^ T cell response in patient #5 suggests WT1 peptide-presentation via MCH class II rather than complex-formation with MHC class I. In support to this hypothesis, Zhang et al. investigated intact proteins and long peptides in the cross-presentation pathway, demonstrating that long peptides traffic to both the endosomes and the cytosol, whereas whole protein was found to traffic only to the endosomal compartments. Consequently, whole proteins could not be processed through the cross-presentation pathway and led to a CD4^+^ T cell restricted response after immunization, while peptide vaccinations also induced a CD8^+^ T cell response [45]. Employing a mouse model, Martins et al. reported on CD4^+^ T cell response being critical for durable vaccine-mediated protection [46]. The lack of humoral response in patient #5 might be based on the concurrent CLL, along with B cell depleting therapies prior to vaccination, while patient #3 demonstrated significant humoral response post-vaccination.

In regard to protein-based vaccination, intracellular processing of the WT1 protein is likely to generate peptides with binding motifs for several HLA molecules. Consequently, no patient selection based on HLA subtype needs to be carried out in clinical application and helps to overcome the need for individualized vaccination strategies.

In line with our safety results, there are hardly any reports about treatment-related toxicity due to WT1 vaccination approaches except mild erythema at the sites of injection. Regarding a systematic review of nine clinical trials with 51 patients [22], solely the trial of Kuball et al. described three patients with grade III toxicity (erythema, dyspnea and fever) after WT1 peptide vaccination [47]. Thus, sole WT1-based vaccination overall had an acceptable safety profile and was well-tolerated which aligns perfectly with our data.

Our study has limitations: while humoral immune response was determined in all five patients, WT1-specific cellular immune response was elucidated only in 1/5 patients, respectively. Levels of anti-WT1 antibodies as well as the emergence of epitope-specific T lymphocytes throughout the study and especially in patient #1 could have helped to further explain the different clinical courses.

Although the number of reported patients is limited and the individual cases are heterogeneous with different genetic risk factors, the clinical outcome exceeded historical published data. To overcome certain caveats, clinical trials with large and homogeneous AML patient cohorts are needed. Nevertheless, our data provide evidence of potential clinical efficacy of WT1 protein-based vaccination therapy in AML patients, thereby supporting future investigations of this immunotherapeutic treatment approach.

## Conclusion

WT1 vaccination therapy showed above-average response durations in three out of five AML patients after induction therapy: one patient demonstrated MRD clearance, another patient showed above-average remission duration and the third patient developed a complete clonal switch at relapse following 18 vaccinations. WT1 protein-based vaccination induced both humoral and cellular immune response and overall had an acceptable safety profile. Markers to predict the likelihood of response still need to be elucidated.

## Supplementary Information

Below is the link to the electronic supplementary material.

Suppl. Figure 1: Immune response of patient #5 (a) Diagrams demonstrating frequency of cytokine positive (TNF+ IFN-γ+ double positive, TNF+ IFN-γ- and TNF- IFN-γ+ single positive) CD8+ T cells measured by flow cytometry. (b) UMAPs showing the total CD3 positive compartment of combined samples. Individual plots are overlaid with the expression of included markers. 1000 cells were subsetted from every sample from each cohort.
Suppl. Figure 2: T cell cytokine profile of patient #5 (a) Representative dot plots of CD3 positive T cells demonstrating expression of tumor necrosis factor (TNF), Interferon-γ (IFNg), granzyme B and perforin.
Suppl. Figure 3: T cell infiltration in the BM. Diagrams demonstrating the frequency of indicated T cell populations of all nucleated precursor cells before and after WT1-based vaccination measured by IHC, shown for (a) patient #2, (b) patient #3 and (c) patient #5.
Supplementary file1 (PDF 480 KB)

Suppl. Table 1: Humoral response: anti-WT1 IgG (EU/ml). Anti-WT1 IgG antibody response measured by ELISA.
Suppl. Table 2: High-dimensional cytometry – surface antibodies
Suppl. Table 3: High-dimensional cytometry – intracellular antibodies
Supplementary file2 (PDF 65 KB)

## Data Availability

All data generated or analyzed during this study are included in this published article and its supplementary information files.

## References

[1] Barrett AJ (2008). Understanding and harnessing the graft-versus-leukaemia effect. Br J Haematol.

[2] Shallis RM, Wang R, Davidoff A, Ma X, Zeidan AM (2019). Epidemiology of acute myeloid leukemia: Recent progress and enduring challenges. Blood Rev.

[3] Papaemmanuil E, Gerstung M, Bullinger L (2016). Genomic Classification and Prognosis in Acute Myeloid Leukemia. N Engl J Med.

[4] Bullinger L, Döhner K, Döhner H (2017). Genomics of acute myeloid leukemia diagnosis and pathways. J Clin Oncol: Off J Am Soci Clin Oncol.

[5] Francke U, Holmes LB, Atkins L, Riccardi VM (1979). Aniridia-Wilms’ tumor association: evidence for specific deletion of 11p13. Cytogenet Genome Res.

[6] Gessler M, Poustka A, Cavenee W, Neve RL, Orkin SH, Bruns GA (1990). Homozygous deletion in Wilms tumours of a zinc-finger gene identified by chromosome jumping. Nature.

[7] Call KM, Glaser T, Ito CY (1990). Isolation and characterization of a zinc finger polypeptide gene at the human chromosome 11 Wilms' tumor locus. Cell.

[8] Yang L, Han Y, Suarez Saiz F, Minden MD (2007). A tumor suppressor and oncogene: the WT1 story. Leukemia.

[9] Menssen HD, Renkl HJ, Rodeck U, Maurer J, Notter M, Schwartz S, Reinhardt R, Thiel E (1995). Presence of Wilms' tumor gene (wt1) transcripts and the WT1 nuclear protein in the majority of human acute leukemias. Leukemia.

[10] Becker H, Marcucci G, Maharry K (2010). Mutations of the Wilms tumor 1 gene (WT1) in older patients with primary cytogenetically normal acute myeloid leukemia: a Cancer and Leukemia Group B study. Blood.

[11] Pronier E, Bowman RL, Ahn J (2018). Genetic and epigenetic evolution as a contributor to WT1-mutant leukemogenesis. Blood.

[12] Galimberti S, Guerrini F, Carulli G, Fazzi R, Palumbo GA, Morabito F, Petrini M (2004). Significant co-expression of WT1 and MDR1 genes in acute myeloid leukemia patients at diagnosis. Eur J Haematol.

[13] Simpson LA, Burwell EA, Thompson KA, Shahnaz S, Chen AR, Loeb DM (2006). The antiapoptotic gene A1/BFL1 is a WT1 target gene that mediates granulocytic differentiation and resistance to chemotherapy. Blood.

[14] Tschan MP, Gullberg U, Shan D, Torbett BE, Fey MF, Tobler A (2008). The hDMP1 tumor suppressor is a new WT1 target in myeloid leukemias. Leukemia.

[15] Vidovic K, Svensson E, Nilsson B, Thuresson B, Olofsson T, Lennartsson A, Gullberg U (2010). Wilms' tumor gene 1 protein represses the expression of the tumor suppressor interferon regulatory factor 8 in human hematopoietic progenitors and in leukemic cells. Leukemia.

[16] Montano G, Ullmark T, Jernmark-Nilsson H, Sodaro G, Drott K, Costanzo P, Vidovic K, Gullberg U (2016). The hematopoietic tumor suppressor interferon regulatory factor 8 (IRF8) is upregulated by the antimetabolite cytarabine in leukemic cells involving the zinc finger protein ZNF224, acting as a cofactor of the Wilms' tumor gene 1 (WT1) protein. Leuk Res.

[17] Paschka P, Marcucci G, Ruppert AS (2008). Wilms' tumor 1 gene mutations independently predict poor outcome in adults with cytogenetically normal acute myeloid leukemia: a cancer and leukemia group B study. J Clin Oncol: Off J Am Soci Clin Onco.

[18] Virappane P, Gale R, Hills R (2008). Mutation of the Wilms' tumor 1 gene is a poor prognostic factor associated with chemotherapy resistance in normal karyotype acute myeloid leukemia: the United Kingdom Medical Research Council Adult Leukaemia Working Party. J Clin Oncol : Off J Am Soci Clin Onco.

[19] Nomdedeu JF, Hoyos M, Carricondo M (2013). Bone marrow WT1 levels at diagnosis, post-induction and post-intensification in adult de novo AML. Leukemia.

[20] Bergmann L, Miething C, Maurer U, Brieger J, Karakas T, Weidmann E, Hoelzer D (1997). High levels of Wilms' tumor gene (wt1) mRNA in acute myeloid leukemias are associated with a worse long-term outcome. Blood.

[21] Ullmark T, Montano G, Gullberg U (2018). DNA and RNA binding by the Wilms' tumour gene 1 (WT1) protein +KTS and -KTS isoforms-From initial observations to recent global genomic analyses. Eur J Haematol.

[22] Di Stasi A, Jimenez AM, Minagawa K, Al-Obaidi M, Rezvani K (2015). Review of the results of WT1 Peptide Vaccination Strategies for Myelodysplastic Syndromes and Acute Myeloid Leukemia from Nine Different Studies. Front Immunol.

[23] Gaiger A, Reese V, Disis ML, Cheever MA (2000). Immunity to WT1 in the animal model and in patients with acute myeloid leukemia. Blood.

[24] Maslak PG, Dao T, Krug LM (2010). Vaccination with synthetic analog peptides derived from WT1 oncoprotein induces T-cell responses in patients with complete remission from acute myeloid leukemia. Blood.

[25] Mailander V, Scheibenbogen C, Thiel E, Letsch A, Blau IW, Keilholz U (2004). Complete remission in a patient with recurrent acute myeloid leukemia induced by vaccination with WT1 peptide in the absence of hematological or renal toxicity. Leukemia.

[26] Keilholz U, Letsch A, Busse A (2009). A clinical and immunologic phase 2 trial of Wilms tumor gene product 1 (WT1) peptide vaccination in patients with AML and MDS. Blood.

[27] Tsuboi A, Oka Y, Kyo T (2012). Long-term WT1 peptide vaccination for patients with acute myeloid leukemia with minimal residual disease. Leukemia.

[28] Yasukawa M, Fujiwara H, Ochi T, Suemori K, Narumi H, Azuma T, Kuzushima K (2009). Clinical efficacy of WT1 peptide vaccination in patients with acute myelogenous leukemia and myelodysplastic syndrome. Am J Hematol.

[29] Maslak PG, Dao T, Bernal Y (2018). Phase 2 trial of a multivalent WT1 peptide vaccine (galinpepimut-S) in acute myeloid leukemia. Blood Adv.

[30] Liu H, Zha Y, Choudhury N (2018). WT1 peptide vaccine in Montanide in contrast to poly ICLC, is able to induce WT1-specific immune response with TCR clonal enrichment in myeloid leukemia. Exp Hematol Oncol.

[31] Nakata J, Nakae Y, Kawakami M (2018). Wilms tumour 1 peptide vaccine as a cure-oriented post-chemotherapy strategy for patients with acute myeloid leukaemia at high risk of relapse. Br J Haematol.

[32] Shirakawa T, Kitagawa K (2018). Antitumor effect of oral cancer vaccine with Bifidobacterium delivering WT1 protein to gut immune system is superior to WT1 peptide vaccine. Hum Vaccin Immunother.

[33] Saylor K, Gillam F, Lohneis T, Zhang C (2020) Designs of Antigen Structure and Composition for Improved Protein-Based Vaccine Efficacy. Frontiers Immunol, 10.3389/fimmu.2020.00283 10.3389/fimmu.2020.00283 PMC7050619 32153587

[34] Cilloni D, Renneville A, Hermitte F (2009). Real-time quantitative polymerase chain reaction detection of minimal residual disease by standardized WT1 assay to enhance risk stratification in acute myeloid leukemia: a European LeukemiaNet study. J Clin Oncol: Off J Am Soci Clin Oncol.

[35] Kreutmair S, Unger S, Nunez NG (2021). Distinct immunological signatures discriminate severe COVID-19 from non-SARS-CoV-2-driven critical pneumonia. Immunity.

[36] Bendall SC, Simonds EF, Qiu P (2011). Single-cell mass cytometry of differential immune and drug responses across a human hematopoietic continuum. Science.

[37] Becht E, McInnes L, Healy J, Dutertre CA, Kwok IWH, Ng LG, Ginhoux F, Newell EW (2018). Dimensionality reduction for visualizing single-cell data using UMAP. Nat Biotechnol.

[38] Van Gassen S, Callebaut B, Van Helden MJ, Lambrecht BN, Demeester P, Dhaene T, Saeys Y (2015). FlowSOM: Using self-organizing maps for visualization and interpretation of cytometry data. Cytometry A.

[39] Döhner H, Estey EH, Amadori S (2010). Diagnosis and management of acute myeloid leukemia in adults: recommendations from an international expert panel, on behalf of the European LeukemiaNet. Blood.

[40] Nagel G, Weber D, Fromm E (2017). Epidemiological, genetic, and clinical characterization by age of newly diagnosed acute myeloid leukemia based on an academic population-based registry study (AMLSG BiO). Ann Hematol.

[41] Oka Y, Tsuboi A, Taguchi T (2004). Induction of WT1 (Wilms' tumor gene)-specific cytotoxic T lymphocytes by WT1 peptide vaccine and the resultant cancer regression. Proc Natl Acad Sci USA.

[42] Rezvani K, Yong AS, Mielke S, Savani BN, Musse L, Superata J, Jafarpour B, Boss C, Barrett AJ (2008). Leukemia-associated antigen-specific T-cell responses following combined PR1 and WT1 peptide vaccination in patients with myeloid malignancies. Blood.

[43] Anguille S, Van de Velde AL, Smits EL, Van Tendeloo VF, Juliusson G, Cools N, Nijs G, Stein B, Lion E, Van Driessche A, Vandenbosch I, Verlinden A, Gadisseur AP, Schroyens WA, Muylle L, Vermeulen K, Maes M-B, Deiteren K, Malfait R, Gostick E, Lammens M, Couttenye MM, Jorens P, Goossens H, Price DA, Ladell K, Oka Y, Fujiki F, Oji Y, Sugiyama H, Berneman ZN (2017). Dendritic cell vaccination as postremission treatment to prevent or delay relapse in acute myeloid leukemia. Blood.

[44] Oka Y, Tsuboi A, Nakata J, Nishida S, Hosen N, Kumanogoh A, Oji Y, Sugiyama H (2017). Wilms' tumor gene 1 (WT1) peptide vaccine therapy for hematological malignancies: from CTL epitope identification to recent progress in clinical studies including a cure-oriented strategy. Oncol Res Treat.

[45] Zhang H, Hong H, Li D (2009). Comparing pooled peptides with intact protein for accessing cross-presentation pathways for protective CD8+ and CD4+ T cells. J Biol Chem.

[46] Martins KAO, Cooper CL, Stronsky SM, Norris SLW, Kwilas SA, Steffens JT, Benko JG, van Tongeren SA, Bavari S (2016). Adjuvant-enhanced CD4 T cell responses are critical to durable vaccine immunity. EBioMedicine.

[47] Kuball J, de Boer K, Wagner E (2011). Pitfalls of vaccinations with WT1-, Proteinase3- and MUC1-derived peptides in combination with MontanideISA51 and CpG7909. Cancer Immun, Immunothera : CII.

