# Supplementary material for: First-in-human study of WT1 recombinant protein vaccination in elderly patients with AML in remission: a single-center experience
Source: Cancer Immunol Immunother. 2022 Apr 27;71(12):2913–28. doi: 10.1007/s00262-022-03202-8 (PMC9588470; doi:10.1007/s00262-022-03202-8)

Suppl Fig 1

a

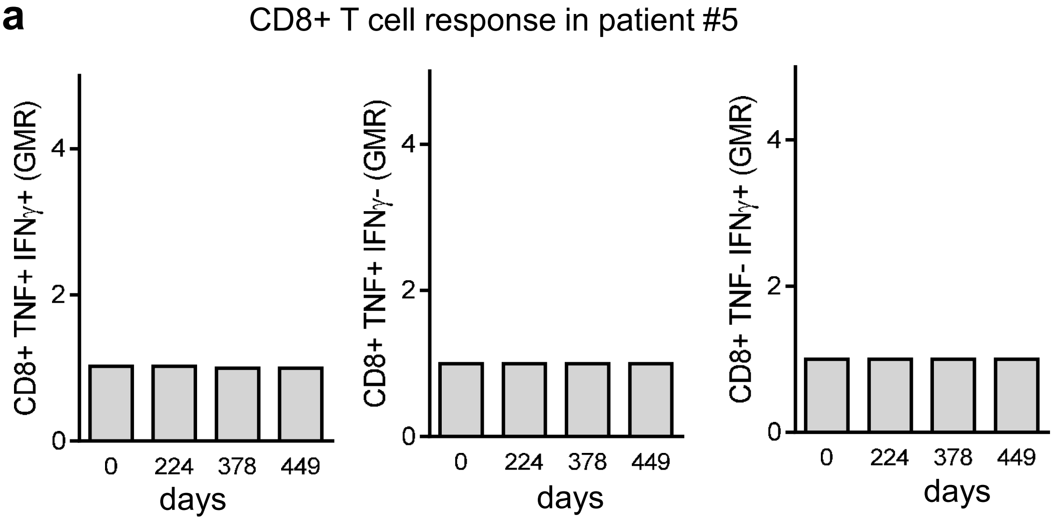

b

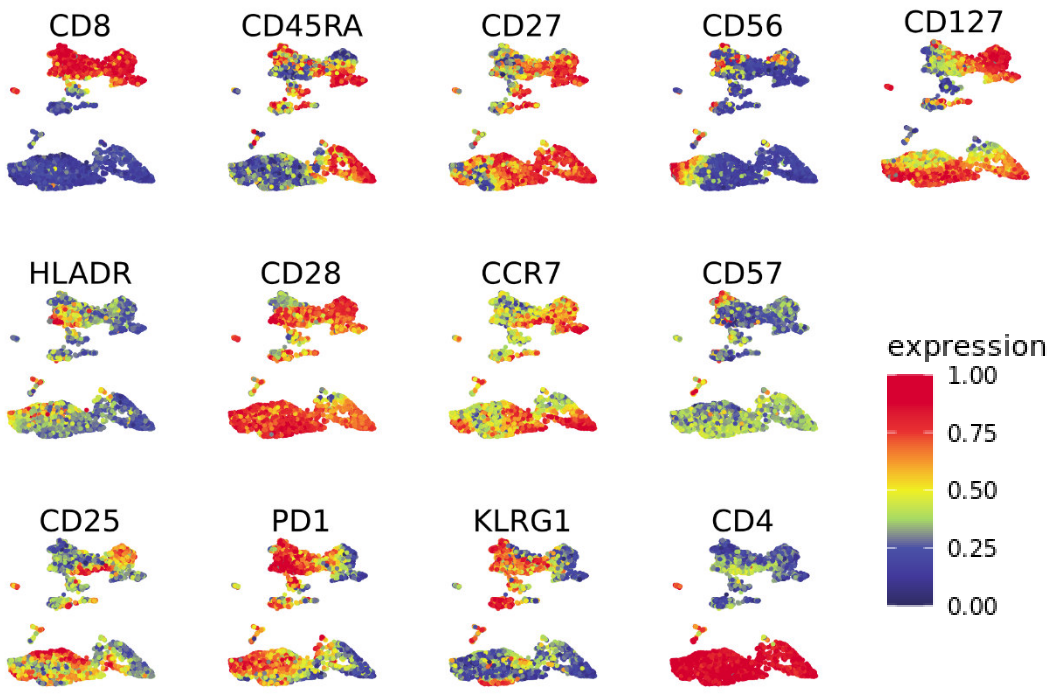

Suppl Fig 2

a T cell cytokine profile in patient #5

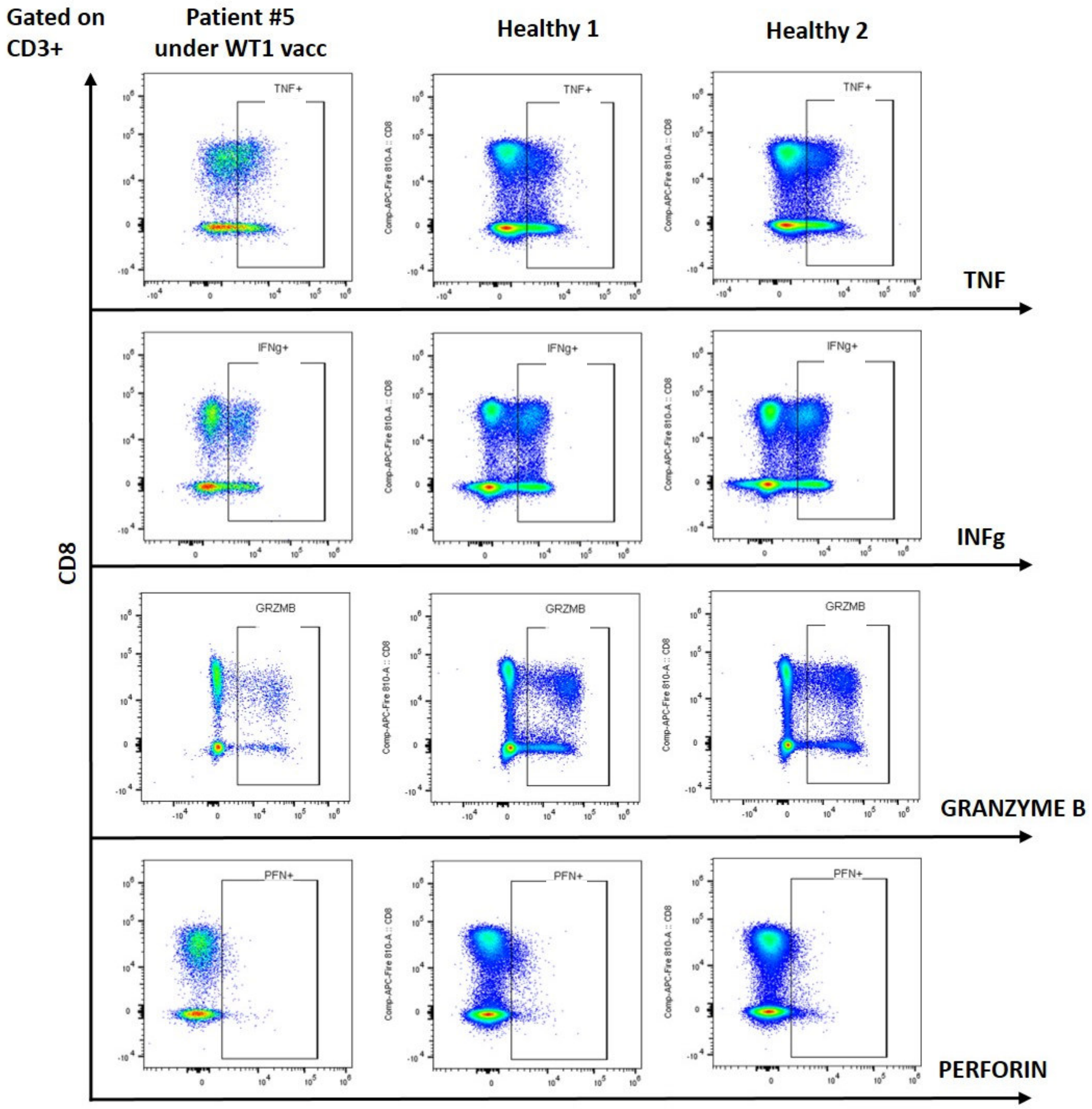

Suppl Fig 3

a

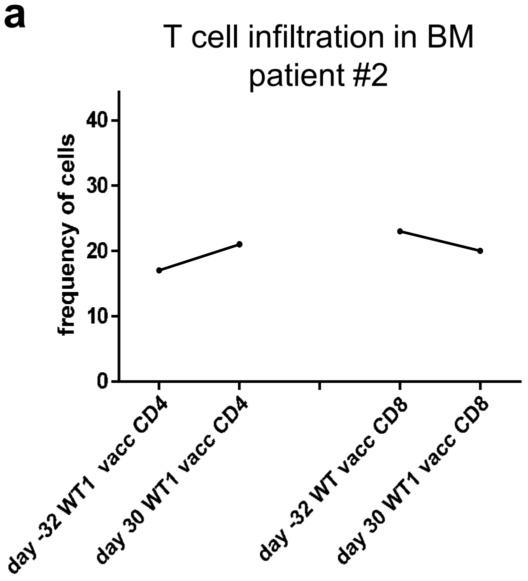

b

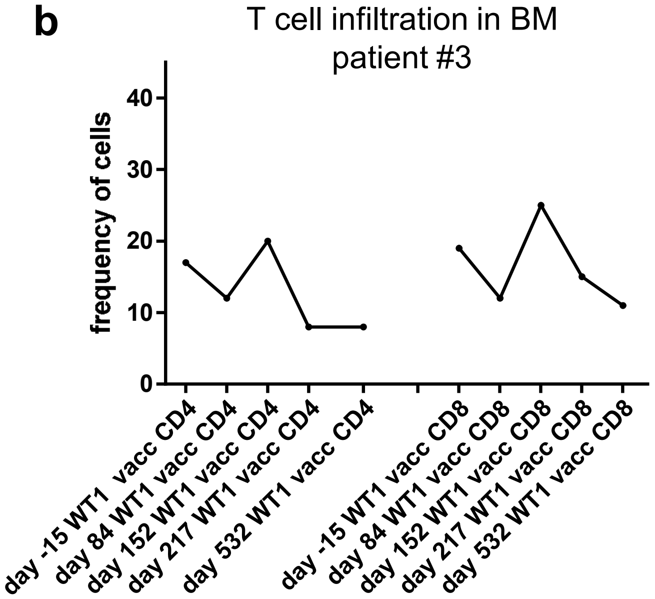

c

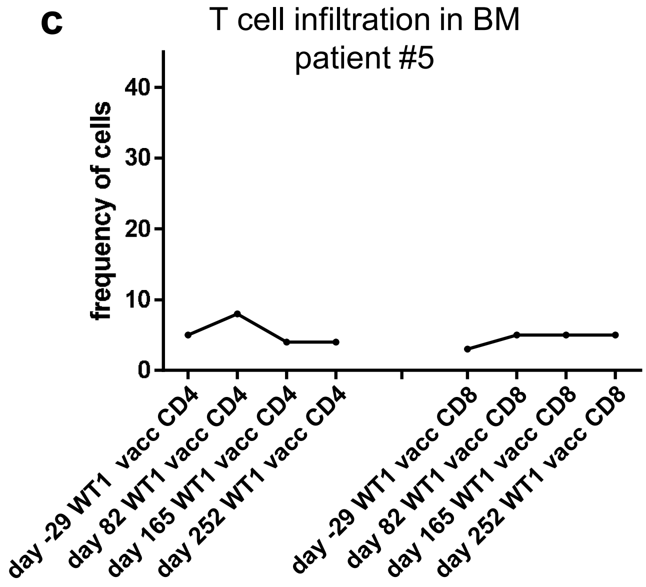

Supplement: Supplementary file 1 — Suppl. Figure 1: Immune response of patient #5 (a) Diagrams demonstrating frequency of cytokine positive (TNF+ IFN-γ+ double positive, TNF+ IFN-γ- and TNF- IFN-γ+ single positive) CD8+ T cells measured by flow cytometry. (b) UMAPs showing the total CD3 positive compartment of combined samples. Individual plots are overlaid with the expression of included markers. 1000 cells were subsetted from every sample from each cohort. Suppl. Figure 2: T cell cytokine profile of patient #5 (a) Representative dot plots of CD3 positive T cells demonstrating expression of tumor necrosis factor (TNF), Interferon-γ (IFNg), granzyme B and perforin. Suppl. Figure 3: T cell infiltration in the BM. Diagrams demonstrating the frequency of indicated T cell populations of all nucleated precursor cells before and after WT1-based vaccination measured by IHC, shown for (a) patient #2, (b) patient #3 and (c) patient #5. Supplementary file1 (PDF 480 KB) [file 262_2022_3202_MOESM1_ESM.pdf]
