# Supplementary material for: First-in-human study of WT1 recombinant protein vaccination in elderly patients with AML in remission: a single-center experience
Source: Cancer Immunol Immunother. 2022 Apr 27;71(12):2913–28. doi: 10.1007/s00262-022-03202-8 (PMC9588470; doi:10.1007/s00262-022-03202-8)
Supplement: Supplementary file 2 — Suppl. Table 1: Humoral response: anti-WT1 IgG (EU/ml). Anti-WT1 IgG antibody response measured by ELISA. Suppl. Table 2: High-dimensional cytometry – surface antibodies Suppl. Table 3: High-dimensional cytometry – intracellular antibodies Supplementary file2 (PDF 65 KB) [file 262_2022_3202_MOESM2_ESM.pdf]

Suppl Table 1 Humoral response: anti-WT1 IgG (EU/ml)

Pat. No.	Prior to vacc.	After 2 vacc. / week 5	After 4 vacc. / week 9	After 6 vacc. / week 13	After 6 vacc. / week 15	After 8 vacc. / week 21	After 12 vacc. / week 32	After 13 vacc./ week 40	After 16 vacc. / week 54	After 17 vacc. / week 64	4 months after last vacc.
**1**	< 7	< 7									
**2**	< 7	< 7									< 7
**3**	< 7	< 7	26	58	50	46					
**4**	< 7	< 7									
**5**	< 7	< 7	< 7	< 7		< 7	< 7	< 7	< 7	< 7	

Suppl Table 2 High-dimensional cytometry – surface staining

Fluorochrome	Antigen	Clone	Company
BUV395	CD45RA	HI100	BD
BUV563	CD27	M-T271	BD
BUV737	CD56	NCAM16.2	BD
BUV805	CD3	UCHT1	BD
BV510	CD3	OKT3	Biolegend
BV570	HLA-DR	L243	Biolegend
BV605	CD28	CD28.2	Biolegend
BV785	CCR7 (CD197)	G043H7	Biolegend
FITC	CD57	HNK-1	Biolegend
Spark Blue	CD4	SK3	Biolegend
PerCP	CD45	2D1	Biolegend
PerCP-efluor710	KLRG1	13F12F2	ThermoScientific
PE-Cy5.5	CD19	SJ25C1	ThermoScientific
PE/Dazzle594	PD-1	EH12.2H7	Biolegend
PE-Cy7	CD25 (IL-2Ra)	M-A251	Biolegend
Spark NIR 685	CD127	A019D5	Biolegend
APC/Fire810	CD8	SK1	Biolegend

Suppl Table 3 High-dimensional cytometry – intracellular staining

Fluorochrome	Antigen	Clone	Company
V450	IFNg	B27	BD
BV750	TNF	Mab11	BD
PerCP-Cy5.5	Perforin	B-D48	Biolegend
AF700	Granzyme B	GB11	BD
